# Gut metabolites predict *Clostridioides difficile* recurrence

**DOI:** 10.1186/s40168-022-01284-1

**Published:** 2022-06-09

**Authors:** Jennifer J. Dawkins, Jessica R. Allegretti, Travis E. Gibson, Emma McClure, Mary Delaney, Lynn Bry, Georg K. Gerber

**Affiliations:** 1 Department of Pathology, Brigham & Woman’s Hospital, Harvard Medical School, Boston, MA USA; 2 Harvard-MIT Health Sciences & Technology, Harvard Medical School, MIT, Cambridge, MA USA; 3 Massachusetts Host-Microbiome Center, Boston, MA USA; 4 Division of Gastroenterology, Brigham & Woman’s Hospital, Harvard Medical School, Boston, MA USA

**Keywords:** Human infection, Longitudinal, Metabolomics, Gastrointestinal, Predictive model, *C. difficile*

## Abstract

**Background:**

*Clostridioides difficile* infection (CDI) is the most common hospital acquired infection in the USA, with recurrence rates > 15%. Although primary CDI has been extensively linked to gut microbial dysbiosis, less is known about the factors that promote or mitigate recurrence. Moreover, previous studies have not shown that microbial abundances in the gut measured by 16S rRNA amplicon sequencing alone can accurately predict CDI recurrence.

**Results:**

We conducted a prospective, longitudinal study of 53 non-immunocompromised participants with primary CDI. Stool sample collection began pre-CDI antibiotic treatment at the time of diagnosis, and continued up to 8 weeks post-antibiotic treatment, with weekly or twice weekly collections. Samples were analyzed using (1) 16S rRNA amplicon sequencing, (2) liquid chromatography/mass-spectrometry metabolomics measuring 1387 annotated metabolites, and (3) short-chain fatty acid profiling. The amplicon sequencing data showed significantly delayed recovery of microbial diversity in recurrent participants, and depletion of key anaerobic taxa at multiple time-points, including *Clostridium* cluster XIVa and IV taxa. The metabolomic data also showed delayed recovery in recurrent participants, and moreover mapped to pathways suggesting distinct functional abnormalities in the microbiome or host, such as decreased microbial deconjugation activity, lowered levels of endocannabinoids, and elevated markers of host cell damage. Further, using predictive statistical/machine learning models, we demonstrated that the metabolomic data, but not the other data sources, can accurately predict future recurrence at 1 week (AUC 0.77 [0.71, 0.86; 95% interval]) and 2 weeks (AUC 0.77 [0.69, 0.85; 95% interval]) post-treatment for primary CDI.

**Conclusions:**

The prospective, longitudinal, and multi-omic nature of our CDI recurrence study allowed us to uncover previously unrecognized dynamics in the microbiome and host presaging recurrence, and, in particular, to elucidate changes in the understudied gut metabolome. Moreover, we demonstrated that a small set of metabolites can accurately predict future recurrence. Our findings have implications for development of diagnostic tests and treatments that could ultimately short-circuit the cycle of CDI recurrence, by providing candidate metabolic biomarkers for diagnostics development, as well as offering insights into the complex microbial and metabolic alterations that are protective or permissive for recurrence.

Video Abstract

**Supplementary Information:**

The online version contains supplementary material available at 10.1186/s40168-022-01284-1.

## Introduction

*Clostridioides difficile* infection (CDI) is the most common cause of healthcare associated infection in the USA, with symptoms ranging from diarrhea to life-threating fulminant colitis [1]. Annually in the USA, there are > 460 K CDI cases and > 30 K deaths, with costs to the health care system estimated at > $4.8 billion [2]. CDI recurrence after initial infection is common, with an estimated overall 15.5% rate of first recurrence, and escalating recurrence risk with each subsequent episode [2, 3]. *Clostridioides difficile* is a Gram positive, anaerobic spore-forming bacteria that can colonize the gut asymptomatically, with estimates of asymptomatic colonization up to 17% of healthy adults in the community and 50% of hospital patients [1, 4]. Toxigenic strains of *C. difficile* can release endotoxins that bind to intestinal epithelial cells to cause cell death and severe inflammation [4, 5]. However, even toxigenic strains have been found to colonize asymptomatically, and dysbiosis of the microbiome is critical for CDI to occur [4]. Indeed, antibiotic exposure, particularly with drugs that deplete gut anaerobes, is a major risk factor for development of CDI [6, 7].

The mechanisms through which gut microbial dysbiosis drives CDI remain incompletely understood, but there is mounting evidence that the gut metabolome plays an important role. *C. difficile* is capable of metabolizing a variety of carbon sources, including proline, glycine, and branched-chain amino acids via Stickland fermentation [8]. Murine studies have shown that CDI decreases amino acid Stickland substrates and increases Stickland products such as 5-aminovalerate, indicating a utilization of Stickland substrates by *C. difficile* [9, 10]. In recent work in gnotobiotic mice, the commensal bacteria *Paraclostridium bifermentans*, which preferentially uses Stickland fermentation for energy and depletes Stickland substrates in the gut, provides strong protection against CDI infection [11]. Certain cholate-derived primary bile acids, which are depleted in a healthy gut microbiome due to microbial metabolism, have been shown to be co-germinants for *C. difficile* in vitro. However, the role of these metabolites *in vivo* is less clear, and recent studies have shown that the mechanism by which microbes such as *Clostridium scindens* provide protection in vivo may be due to their utilization of *C. difficile’s* preferred carbon sources, rather than through primary bile acid depletion [11–13]. Short chain fatty acids (SCFAs) have also been associated with CDI, although their role is less clear. Acetate and butyrate, gut microbial products of dietary fiber fermentation, have been associated with general gut health in some studies; butyrate, in particular, is a primary energy source for colonocytes and thus may help maintain intestinal barrier integrity [14]. However, *Clostridium sardiniense*, which significantly increases butyrate in the gut, was not protective against CDI in gnotobiotic animal studies, and in fact worsened infection [11]. Taken together, evidence drawn from in vitro or murine studies suggests that CDI may be driven by a multifactorial gut metabolic dysbiosis, which includes alterations in carbon sources.

Despite compelling evidence for the importance of gut metabolomic dysbiosis in CDI, to our knowledge, there have only been three studies that analyzed metabolic factors of CDI in reasonably sized (> 20 subjects) human cohorts. Allegretti et al. performed a cross-sectional comparison of bile acid profiles of participants with first-time CDI (*n* = 20), recurrent CDI (*n* = 19), and no CDI (*n* = 21), and found higher primary bile acids and lower secondary bile acids in those with CDI versus those without CDI [15]. Robinson et al performed a cross-sectional analysis of untargeted metabolomes of participants (*n* = 186) with CDI versus with non-CDI diarrhea, and found higher Stickland fermentation products and lower fructose in CDI participants [16]. Bushman et al. compared the metabolomes of children with IBD (*n* = 27), children with IBD and CDI (*n* = 23), and healthy controls (*n* = 38) at CDI diagnosis, 4 weeks, and 8 weeks later, and found higher primary bile acids, sphingomyelins, and intracellular fatty acids in children with CDI + IBD and in children with IBD and no CDI [17].

CDI recurrence has also been relatively understudied, and it remains unclear whether the metabolic factors described above for primary CDI play similar roles in recurrent disease. A few studies have investigated the role of gut microbiome composition in CDI recurrence. Khanna et al. used 16S rRNA gene amplicon sequencing to analyze the fecal microbiomes of 88 participants at initial CDI diagnosis, and did not find any significant difference in alpha or beta diversity between recurrers and non-recurrers [18]. Seekatz et al. followed 93 participants with initial CDI longitudinally over a range of 1–800 days (to assess both recurrence and re-infection), performed 16S rRNA gene sequencing on samples, and found that alpha diversity trended lower in recurrers [19]. Pakpour et al. formally assessed whether the composition of the gut microbiome could predict recurrence, but found only a weak relationship (area under the receiver-operator curve [AUC] of 0.61) [20]. Four other studies have investigated predicting recurrence solely using electronic health record (EHR) data, and have achieved AUCs ranging from 0.67 to 0.82 [21–24]. Three of these studies found proton-pump inhibitor use to be predictive of recurrence, and two of the studies found higher age to be predictive of recurrence; however, there were no other predictive features common among the studies. Moreover, validation of two of these studies on independent cohorts was attempted and found poor predictive accuracy [25].

To address the gaps in prior studies, including cross-sectional analyses, lack of metabolomic data, and potentially confounding comorbidities or antibiotic use, we conducted a prospective, longitudinal study of participants recruited consecutively from the inpatient service at the Brigham and Women’s Hospital (BWH) and two affiliated hospitals. Exclusion criteria included inflammatory bowel disease, inherited or acquired immunodeficiencies, severe or fulminant CDI, or ongoing non-CDI antibiotic use that continued past the CDI antibiotic course. Participants were followed for up to 8 weeks after completion of their CDI antibiotic treatment or until recurrence. We previously reported an analysis of the clinical and demographic characteristics of the full cohort of 75 participants, 22 of whom experienced recurrent CDI [23]. In the present study, we report on detailed multi-omic analyses of serial stool samples from 53 participants (with 19 recurrences) from the parent study, using broad LC/MS metabolomic profiling, 16S rRNA amplicon sequencing, and targeted short-chain-fatty-acid (SCFA) analysis. We use univariate and multivariate statistical techniques to investigate how microbial composition and metabolomes of recurrers vs. non-recurrers changed and diverged over time. Further, we use cross-validated machine learning/statistical methods to quantify the capability of the data sources to predict future recurrences.

## Results

### Longitudinal study of recurrent CDI measuring gut microbial composition and metabolome

**Fig. 1:**
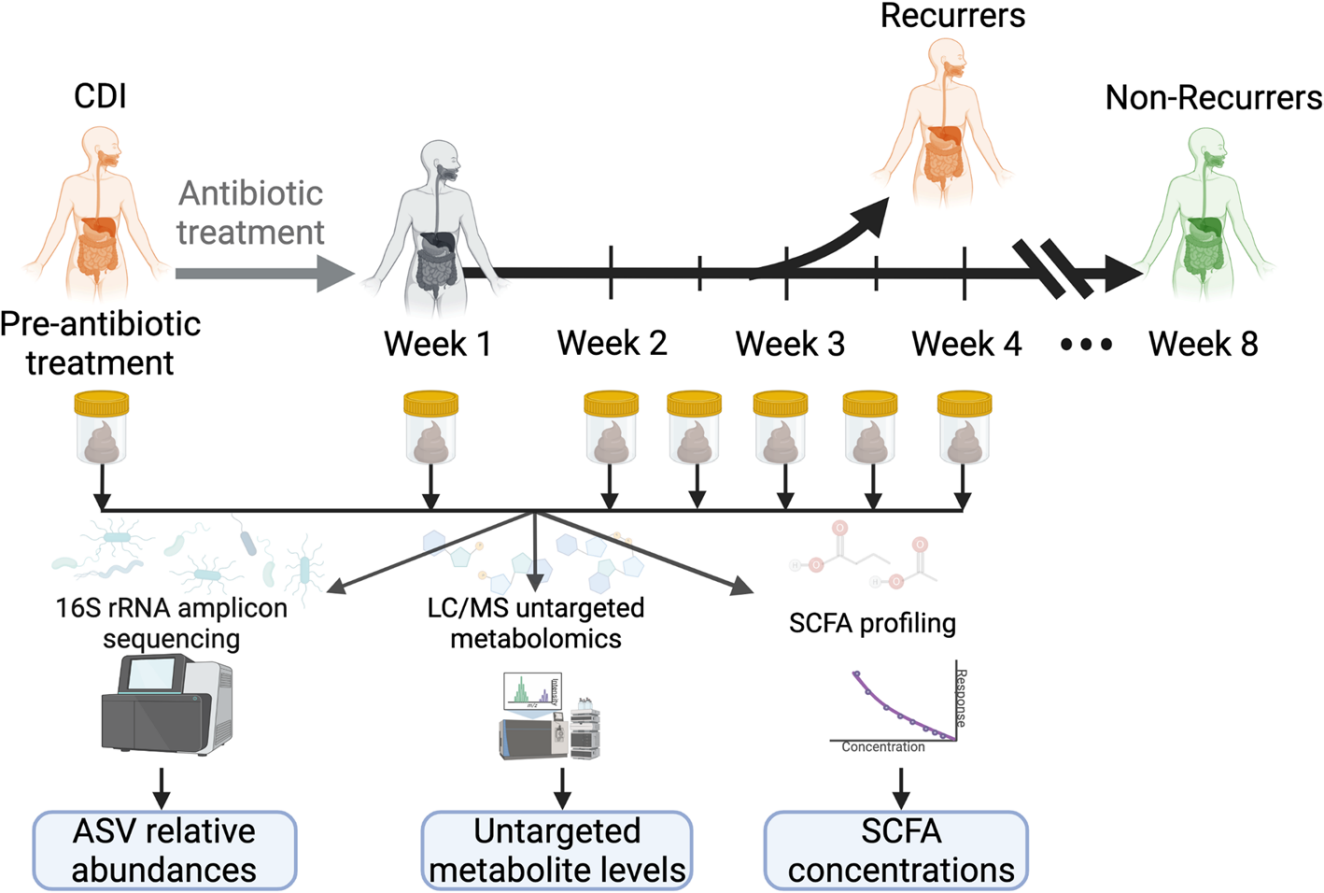
Prospective *Clostridioides difficile* infection (CDI) study measuring gut microbes and metabolites to develop recurrence predictors. Fifty-three participants with first-time CDI were followed for up to 8 weeks after initial CDI antibiotic treatment. Fecal samples were collected prior to CDI antibiotic treatment, 1 week post-treatment, and then weekly or bi-weekly until recurrence or end of the study period. Microbial composition within fecal samples was analyzed with 16s rRNA gene amplicon sequencing. Metabolites in fecal samples were measured with liquid chromatography/mass spectrometry (LC/MS) broad metabolomics and targeted short chain fatty acid profiling

We performed multi-omic analyses on serial fecal samples (Fig. 1) from 53 subjects who had participated in a parent prospective, longitudinal study of CDI at Brigham and Women’s Hospital (BWH) and two affiliated hospitals [23]. In the parent study, fecal samples were collected at the time of diagnosis (if available), 1 week after antibiotic treatment, and every week or half week for up to 8 weeks. Participants for the multi-omic study were chosen from the parent study based on the availability of a week 1 stool sample, a desired ratio of approximately 2:1 non-recurrers to recurrers to sufficiently power predictive analyses while maximizing study resources, and age and sex matching between non-recurrers and recurrers. This yielded a cohort of 34 non-recurrent and 19 recurrent participants for the multi-omic study. Table 1 provides demographic and clinical data for this cohort. Comorbidities for participants are provided in Additional file 1. The only demographic or clinical variable found to be significantly associated with CDI recurrence was the use of enzyme immunoassay (EIA) testing vs. PCR for initial CDI diagnosis (*p* = 0.046). This finding is consistent with analyses of the parent study, and may be due to higher-false positive rates or detection of non-toxin elaborating *C. difficile* strains with the PCR test. The parent study also found treatment of initial CDI with metronidazole versus vancomycin to be associated with recurrence; this association was only borderline significant (*p* = 0.12) in the subset of patients analyzed for our multi-omic study, likely due to the smaller sample size. Note that while the parent study found a significant association between platelet count and CDI recurrence, we were not able to analyze this variable for the subset of participants in our multi-omic study, due to missing blood work data in > 25% of these participants.

**Table 1:** Participant demographic and clinical data

	Recurrers *N* = 19	Non-recurrers *N* = 34	***P*** value
**Demographic data**			
**Race**			
*Black*	1	7	0.36
*Hispanic*	3	2	
*White*	15	25	
**Sex**			
*Male*	5	13	0.55
*Female*	14	21	
**Age**			
*Mean*	57.2 ± 17.2	58.4 ± 14.3	0.7
*Range*	(22, 93)	(30, 87)	
**Clinical data**			
**Body-mass index (BMI)**			
*Mean*	28.3 ± 6.9	29.2 ± 9.3	0.87
*Range*	(20.1, 45.1)	(19.4, 66.7)	
**Test used for diagnosis**			
*PCR*	4	17	0.046
*EIA toxin*	15	17	
**Initial treatment antibiotic used**			
*Vancomycin*	11	27	0.12
*Metronidazole*	8	7	
**Prior PPI use**			
*No*	11	17	0.77
*Yes*	8	17	

Statistical testing was performed using Fisher’s exact test for binary variables, the chi-squared test for categorical variables, and the Wilcoxon rank-sum test for continuous variables

*PCR* Polymerase chain reaction, *GDH* Glutamate dehydrogenase, *PPI* Proton-pump inhibitor

Table 2 provides a summary of the fecal samples analyzed. Because all participants who recurred did so within the first 3 weeks after initial treatment, we focused our subsequent analyses primarily on data-points prior to week 3. This time window provides a sufficient number of recurrent samples for statistical testing and also, in the context of developing diagnostic testing in the future, represents a relevant time period for clinically actionable decision-making. Each sample was analyzed with (1) 16S rRNA amplicon sequencing, (2) liquid-chromatography/mass-spectrometry (LC/MS) untargeted metabolomics, and (3) targeted short chain fatty acid (SCFA) analyses. For amplicon analyses, this yielded 4,605,740 total sequencing reads (average of ~ 10 K/reads per sample) and subsequent bioinformatic processing with dada2 produced 2509 unique amplicon sequence variants (ASVs). The LC/MS untargeted metabolomics platform quantified 1387 unique and annotated metabolites. SCFA analyses quantified nine metabolites: acetate, propionate, isobutyrate, butyrate, isovalerate/2-methylbutyrate (indistinguishable by the platform used), valerate, isocaproate, caproate, and heptanoate. However, heptanoate and caproate were only present in one or two samples, respectively, and were thus removed from subsequent analyses.

**Table 2:** Number of samples analyzed for each time-point

Week	Non-recurrers	Recurrers
**Pre-antibiotics**	18	8
**1**	34	14
**1.5**	10	3
**2**	34	6
**2.5**	10	2
**3**	33	3
**3.5**	4	0
**4**	34	0

Note that five participants recurred during week 1, so their samples were not included in week 1 analyses

### Participants who recurred exhibited slower recovery of gut microbiome diversity and composition post-CDI antibiotic treatment

To gain high-level insights into the structure of microbiomes, we assessed their overall ecological diversity using alpha [26] and beta diversity measures [27]. For more detailed understanding of microbiomes, we analyzed taxonomic composition at the level of amplicon sequence variants (ASVs). After filtering low abundance/rare taxa, we obtained 237 ASVs, which we used for subsequent DESeq2 fold-change analyses [28]. Because participants received treatment for initial CDI with either vancomycin or metronidazole, and these antibiotics are known to have differential effects on the gut microbiome, we included terms in DESeq regression models to account for the antibiotic type used, and report only changes that remained significant when controlling for the antibiotic type. For analyses at both the level of ecological diversity and at the level of ASVs, we performed both intra-group (i.e., differences in diversity or abundance of ASVs between time-points within the same group, either non-recurrers or recurrers) and inter-group (differences in diversity or abundance of ASVs between non-recurrers and recurrers).

**Fig. 2:**
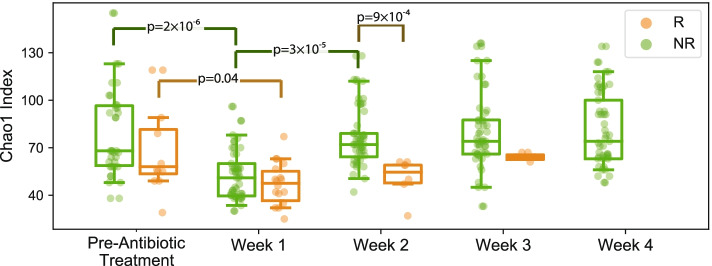
Ecological diversity of recurrers’ gut microbiomes recovered significantly more slowly than non-recurrers’. Alpha diversity (Chao index), a measure of species richness, significantly decreased pre- to 1 week post-CDI antibiotic treatment within both recurrent and non-recurrent groups. From one week to two weeks post-CDI treatment, alpha diversity recovered significantly only within the non-recurrent group. Alpha diversity only differed significantly between the recurrent and non-recurrent groups at 2 weeks post-CDI antibiotic treatment, with higher diversity in the non-recurrent group. *R* = recurrers, NR = non-recurrers

We first investigated intra-group diversity changes, comparing pre- versus 1 week post-CDI antibiotic diversity in either recurrers or non-recurrers. For these comparisons, alpha diversity significantly decreased within both recurrent (*p* = 0.04) and non-recurrent (*p* = 2 × 10^−4^) groups (Fig. 2; Additional file 2), consistent with depletion of gut microbes during antibiotic treatment for CDI. We evaluated intra-group beta diversity using the Bray-Curtis dissimilarity measure and found a similar pattern (Fig. S1): significant changes were seen in both recurrers (*p* = 5 × 10^−3^) and non-recurrers (*p* = 10^−3^). We next evaluated intra-group diversity from week 1 to week 2, and interestingly found that both alpha and beta diversity recovered significantly only within the non-recurrent group (*p* = 3 × 10^−5^, and *p* = 10^−3^ respectively (Additional file 2).

Comparing inter-group alpha between the recurrent and non-recurrent groups at each time-point, we found a significant difference in alpha diversity at week 2 post-CDI antibiotic treatment, with higher diversity in the non-recurrent group (*p* = 9 × 10^−4^) (Fig. 2, Additional file 2). We did not find significant inter-group differences in beta diversity. Taken together, these intra- and inter-group diversity analyses suggest that recurrent and non-recurrent participants both had expected declines in gut microbiome ecological diversity with antibiotic treatment for CDI, but recurrent subjects exhibited delayed recovery of microbial diversity.

We next examined intra-group differences in ASV abundances over time using differential abundance analysis. In non-recurrers, 30 ASVs significantly differed in abundance between week 1 to week 2. Among these 30 ASVs, 25 exhibited significant increases (Additional file 3). Of these ASVs, 15 were in the Lachnospiraceae family, representing a significant enrichment for this bacterial family (FDR = 0.003, Additional file 4), which are generally strict anaerobes with specialized niches and associated with normal microbiome function. In recurrers, 13 ASVs were significantly different in abundance from week 1 to week 2, with 8 of these exhibiting significant increases. This set of ASVs did not demonstrate significant enrichment for any particular taxa, and, in contrast to changes seen in non-recurrers, only one ASV exhibiting significant increases from week 1 to week 2 was from the Lachnospiraceae family (ASV 76) (Additional file 3).

**Fig. 3:**
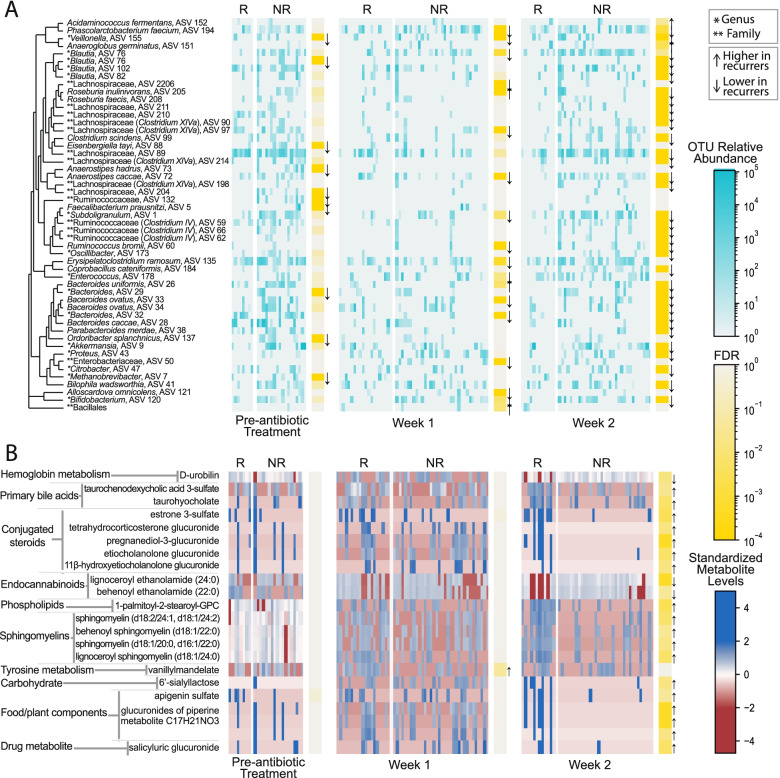
Gut microbiome taxa and metabolite levels differed significantly between CDI recurrent and non-recurrent participants. **A** Univariate analyses of 16S rRNA gene amplicon sequencing data found 51 out of 237 amplicon sequencing variants (ASVs) (post-filtering to remove rare or low-variance taxa), were significantly differentially abundant between recurrers versus non-recurrers. **B** Univariate analyses of LC/MS untargeted metabolomics found 22 out of 1387 metabolites (post-filtering to remove rare or low-variance metabolites), were significantly differentially abundant between recurrers versus non-recurrers. Metabolite levels shown are log-transformed and standardized. R = recurrers, NR = non-recurrers. Arrows denote the direction of the statistically significant effect. Participants (columns) were ordered in the figure via hierarchical clustering

Finally, we assessed inter-group differences in ASV composition, comparing between non-recurrers and recurrers at pre-CDI treatment, week 1, or week 2 post-CDI treatment. This analysis showed that non-recurrers had significantly higher abundances of 10 ASVs pre-CDI treatment, 15 ASVs at week 1 post-CDI treatment, and 35 ASVs at week 2 (Fig. 3A; Additional file 3). The set of ASVs at increased abundance at week 2 was significantly enriched for taxa in the Bacteroidaceae (FDR = 0.03), Ruminococcaceae (FDR = 0.03), and Lachnospiraceae (FDR = 0.03) families (Additional file 4). Many of the taxa in these families found to be significantly increased in non-recurrers have been associated with normal microbiome function, including *Clostridium* cluster XIVa taxa (ASVs 90, 97, 99, 198, 214) within the Lachnospiraceae family and *Clostridium* cluster IV taxa (ASVs 59, 60, 62, 66) within the Ruminococcaceae family [29] (Additional file 3). Interestingly, one of the *Clostridium* cluster XIVa taxa at higher abundance in non-recurrers (significant at week 2 and with a trend toward higher abundance at other time-points) was *Clostridium scindens* (ASV 99), which has been shown to provide host resistance to *C. difficile* [12, 13]. A number of the other genera found to be at higher abundance in non-recurrers have been previously linked to protection against CDI in human studies, including *Bacteroides* (ASVs 26, 28, 29, 32, 33, 34) and *Veillonella* (ASV 154) [20, 30]. Taken together, these intra- and inter-group comparisons of taxa abundances suggest a picture of broader depletions of the normal microbiome in recurrers, evident even pre-CDI antibiotic treatment, but with increasingly more pronounced differences over time, consistent with slower recovery of recurrers’ microbiomes.

### Participants who recurred exhibited an altered gut metabolome indicative of reduced gut microbiome function, and host inflammation and reduced immune modulatory capabilities

We first performed ordination analyses to evaluate overall changes and differences in broad gut metabolomic profiles between recurrers and non-recurrers (Fig. S2). Paralleling our findings on changes in microbial diversity, ordination analyses on metabolomic data (760 metabolites after filtering) showed that the metabolomes of non-recurrers changed significantly from pre-CDI treatment to week 1 post-CDI treatment (*p* = 10^−3^), and from week 1 to week 2 post-CDI treatment (*p* = 10^−3^), but the metabolomes of recurrers only changed significantly from pre-CDI treatment to week 1 post-CDI treatment (*p* = 10^−3^) (Additional file 2). Comparing recurrers to non-recurrers at each time-point, differences were only significant at week 2 (*p* = 0.001), which recapitulated our findings in microbiome alpha diversity (Additional file 2). Taken together, we saw parallel patterns for overall gut metabolomic profiles and microbial diversity, with recurrers and non-recurrers initially exhibiting similar gut metabolomes that only significantly diverged by week 2 post-CDI antibiotic treatment, due to a slower recovery in the recurrent group.

To determine which gut metabolites contributed to these overall patterns of metabolome recovery or non-recovery, we performed univariate analyses (controlling for the antibiotic used for CDI treatment, as described above), both across time and between recurrers and non-recurrers on broad metabolomic data (Fig. 3B, Additional file 3) and targeted SCFA data (Fig. S3, Additional file 3). Changes in metabolites from week 1 to week 2 were significant only for non-recurrers, with 131 metabolites significantly changing over the week. These metabolites were borderline significantly enriched for secondary bile acids (FDR = 0.06), primary bile acids (FDR = 0.06), and hydroxy acyl carnitines (FDR = 0.06), corticosteroids (FDR = 0.07), food components (FDR = 0.07), and disaccharides/oligosaccharides (FDR = 0.10) (Additional file 4). Changes in SCFAs from week 1 to week 2 (Additional file 3) were similarly only significant for non-recurrers, with six SCFAs significantly higher in week 2: acetate (FDR = 6 × 10^−5^), isovalerate/2-methylbutyrate (FDR = 4 × 10^−4^), butyrate (FDR = 10^−3^), valerate (FDR = 0.03), and isobutyrate (FDR = 0.03). Comparison between levels of gut metabolites in recurrers versus non-recurrers showed increasing differences over the study. At pre-treatment, no metabolites were found to be significantly different between recurrers and non-recurrers. However, at week 1 post-CDI treatment, vanillylmandelate (FDR = 0.05) was found to be significantly higher in recurrers. At week 2, abundances of 21 metabolites differed significantly between recurrers and non-recurrers (Additional file 3), with 18 of these metabolites showing higher levels in recurrers. This pattern of increasing divergence over time between gut metabolomes of recurrers and non-recurrers parallels the pattern seen with microbiome composition, suggesting slower recovery of the gut metabolome in recurrers.

The specific changes or differences in metabolites observed can generally be organized into three categories indicative of (1) host inflammation or intestinal damage, (2) lack of microbial deconjugation activity, (3) host alterations in immune and inflammatory capabilities. Vanillylmandelate (VMA), higher in recurrers at week 1 post-CDI treatment, is an end product of catecholamine metabolism and has been previously reported as a biomarker of inflammation [31]. By week 2 post-CDI treatment, biomarkers of cell death were significantly elevated in recurrers. The overall set of metabolites differentiating recurrers and non-recurrers at week 2 was significantly enriched for sphingomyelins (FDR = 7 × 10^−4^, Additional file 4), including lignoceroyl sphingomyelin d18:1/24:0, sphingomyelin d18:2/24:1, d18:1/24:2, sphingomyelin d18:1/20:0, d16:1/22:0, and behenoyl sphingomyelin d18:1/22:0. In additional to sphingomyelins, the phospholipid palmitoyl-2-stearoyl-GPC 16:0/18:0 was also significantly higher in recurrers. Elevated sphingomyelin and phospholipid metabolites have previously been associated with active intestinal epithelial damage, such as in murine models of CDI and in humans with CDI or IBD [10, 17].

At week 2, evidence of impaired microbial function in the gut was also present in recurrers’ metabolomes. Glucuronide and sulfate conjugates were significantly higher in recurrers, including five steroid conjugates (pregnanediol-3-glucorinide, estrone 3-sulfate, 11 beta-hydroxyetiocholanolone glucuronide, etiocholanolone glucuronide, and tetrahydrocorticosterone glucuronide) and four additional glucuronidated compounds (three glucuronides of piperine metabolite C17H21NO3 and salicyluric glucuronide). Gut microbes are critical for deconjugation activities [32, 33]; thus, elevated levels of conjugated metabolites in recurrers may indicate significantly blunted recovery of this normal microbiome function. The microbiome is also critical for transforming bile acids. Indeed, two bile acids, taurocholate (FDR = 0.006) (a primary bile acid) and taurochenodeoxycholic acid 3-sulfate (FDR = 0.03) (a primary bile acid conjugate), were significantly higher in recurrers, again suggesting delayed recovery of microbiome function. Interestingly, taurocholate and other cholate derivatives have been demonstrated to promote *C. difficile* germination in vivo, although their role in pathogenesis in vitro is less clear [1, 11, 13, 34, 35]. Bilirubin metabolism is another major function of the gut microbiota [36]. D-urobilin, the end product of bilirubin metabolism, was significantly lower in recurrers, also suggesting a lack of recovery of microbiome function [FDR = 0.01]. Higher levels of SCFAs indicate active microbiota metabolism in the gut [14]. Consistent with the picture of gut microbial dysbiosis seen with the other metabolites discussed above, levels of acetate (FDR = 0.07) and isovalerate/2-methylbutyrate (FDR = 0.07) were borderline significant for being lower in recurrers.

At week 2, levels of metabolites involved in host immune or inflammatory modulation, predominately conjugated anti-inflammatory compounds and endocannabinoids, also differed significantly between recurrers and non-recurrers. The observed lower levels of conjugated corticosteroids in non-recurrers not only indicates greater microbial deconjugation activities, but may also indicate increased host anti-inflammatory activity: unconjugated corticosteroids, such as tetrahydrocorticosterone, are key anti-inflammatory compounds, and unconjugated sex steroids have also been shown to act as important modulators of inflammation in the gut [33, 37]. Other conjugated compounds found to be significantly higher in recurrers, specifically glucuronides of piperine, salicyluric glucuronide, and apigenin sulfate, have also been shown to have unconjugated forms with anti-inflammatory effects [38–41]. Levels of the endocannabinoids behenoyl ethanolamide (FDR = 5×10^−4^) and lignoceroyl ethanolamide (FDR = 0.04) were significantly lower in recurrers. Endocannabinoids have been shown to maintain gut homeostasis through modulating the immune system and gut motility; additionally, endocannabinoids have been found to increase in the presence of *Akkermansia muciniphila*, a taxa we found to be significantly more abundant in non-recurrers at week 2 (ASV 9, FDR = 2 × 10^−19^) [42–44]. Taken together, these results suggest a picture of reduced capability to modulate inflammation in recurrers.

### Predictive models of recurrence achieved highest accuracy using metabolomic data

To estimate how well our data can predict CDI recurrence in patients, we built supervised machine learning/statistical models and evaluated them using cross-validation. This approach fundamentally differs from the univariate statistical tests presented in the previous sections in two ways: (1) univariate approaches evaluate one variable at a time, and thus cannot find combined effects (e.g., increased risk if multiple metabolites are elevated), and (2) statistical testing approaches cannot provide an estimate of predictive accuracy, or how well the model might perform on unseen data. Both these capabilities are necessary for developing a clinically useful diagnostic, which is an important objective in the field. For prediction tasks, we evaluated three standard methods: lasso-logistic regression (LR), random forests (RF), and lasso-Cox regression (CR). The first two methods predict binary outcomes (recurrence or non-recurrence), whereas CR predicts the time to recurrence. We evaluated these methods based on their ability to predict outcomes using a cross-validation methodology (training the models on subsets of the data and predicting on held-out data). For the two methods predicting binary outcomes, we used the area under the receiver operator curve (AUC) score as the evaluation metric, and for CR we used the concordance index (CI).

**Fig. 4:**
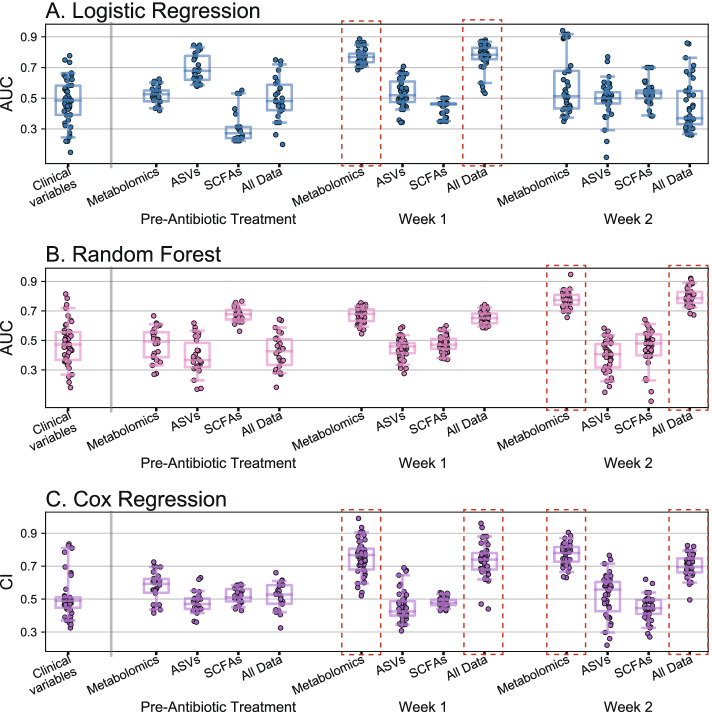
Predictive modeling of CDI recurrence achieved the highest accuracy using metabolomic data. The performance of predictive models was assessed using leave-one-out cross-validation (*N* = 26 at pre-CDI treatment, *N* = 48 at week 1, and *N* = 40 at week 2). Data sources input to models were (1) clinical variables associated with recurrence in prior studies (age, previous PPI use, antibiotic treatment regimen, and CDI diagnostic test used), (2) untargeted gut metabolomics, (3) amplicon sequencing variants (ASVs) of the gut microbiome, (4) gut short-chain fatty acids (SCFAs), (5) data sources 1–4 combined. Performance of **A** logistic regression with lasso and **B** random forests, which predict binary labels (recurrence/no recurrence), were assessed with the area-under-the-curve (AUC) metric. **C** Cox regression, which predicts survival time, was assessed with the concordance index (CI). Models achieving median ≥ 0.70 AUC or CI scores (adequate performance) are denoted with red dashed rectangles. The “All Data” models with ≥ 0.70 AUC or CI were found to select only metabolomic features

We applied LR, RF, and CR to participants’ pre-CDI treatment, week 1, and week 2 samples, using the following information: (1) clinical variables found to be associated with recurrence in prior studies (age [22, 24], previous PPI use [21, 22, 24], treatment regimen [23], and diagnosis method [23]), (2) ASVs from 16S rRNA amplicon sequencing, (3) LC/MS untargeted metabolomics, (4) SCFAs, and, (5) all sources of data (1–4) combined. Overall, we found that the LC/MS metabolomic data at weeks 1 and 2 had the highest predictive accuracy (Fig. 4; Additional file 5). For predicting recurrence/non-recurrence, at week 1, LR on metabolomic data achieved the highest AUC (0.77; [0.71, 0.86] 95% interval), and at week 2, RF on metabolomic data achieved the highest AUC (0.77; [0.69, 0.85] 95% interval). None of the other data sources or time-points achieved AUC scores greater than 0.7, which is generally considered the threshold for an acceptable clinical test (with 0.8 to 0.9 considered excellent). Models predicting recurrence using all available data sources combined achieved essentially equivalent AUCs to models using only metabolomics data (Fig. 4); moreover, these models only consistently selected metabolites as the significant features needed to make predictions (Additional file 6). Prediction of survival time using CR followed similar trends, as all models that achieved CIs > 0.7 selected only metabolites to make predictions. Both ASVs and SCFAs at pre-CDI treatment achieved median AUCs close to 0.7 (0.68 using LR for ASVs, and 0.68 using RF for SCFAs). However, the 95% cross-validated intervals for these AUCs were large, with their lower ranges extending toward values near 0.5 (random chance). Thus, these predictors lack robustness or generalizability. The lack of accurate pre-treatment predictors may have been limited by sample sizes in our study, as fewer samples were available pre-CDI treatment (*N* = 26), compared to weeks 1 and 2 (*N* = 48 and *N* = 40, respectively).

**Fig. 5:**
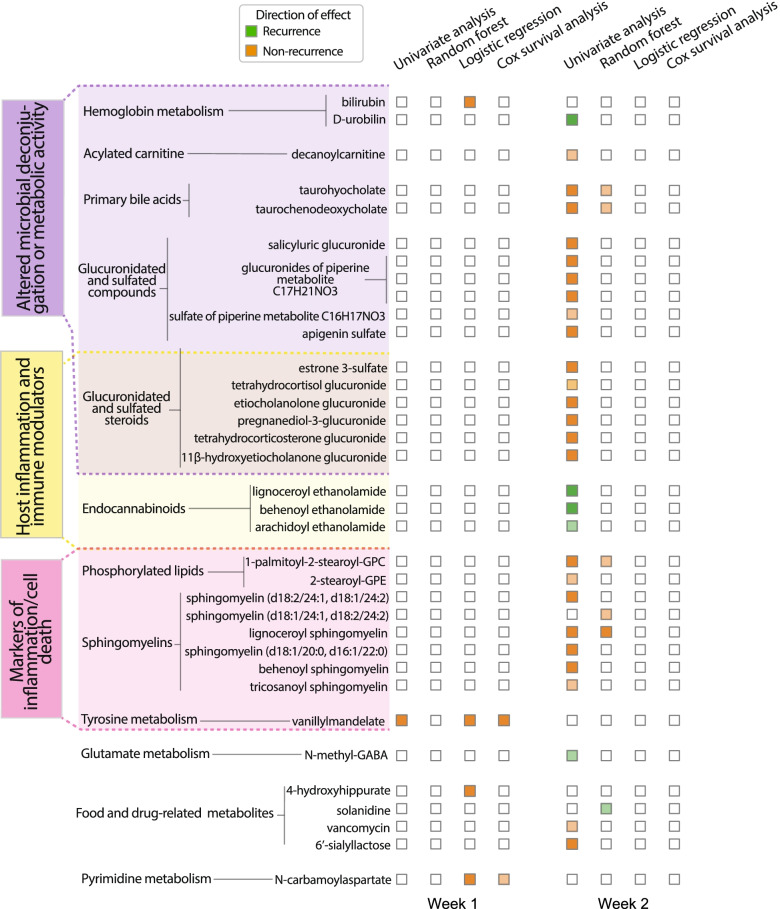
Multiple predictive methods revealed pre-recurrence metabolic alterations mapping to host or microbiome-associated processes. Thirty-seven metabolites were significant in at least one analysis method for distinguishing CDI recurrent versus non-current status. These metabolites fell into one of three categories, reflecting altered host or microbiome activities. Dark orange or green colors indicate significance (FDR < 0.05 in univariate analyses; 95% cross-validated log-odds/feature importance interval not containing 1.0 for predictive models). Light orange or green colors indicate borderline significance (0.05 < FDR ≤ 0.10 in univariate analyses; 75% log-odds/feature importance cross-validated interval not containing 1.0 for predictive models)

To determine which metabolites were predictive in models with median AUCs > 0.7, we assessed cross-validated odds ratios and Gini feature importance measures. At week 1, LR, RF, and CR all selected N-carbamoylaspartate and vanillylmandelate as the top predictors, both of which favored recurrence when at higher levels (Fig. 5). Of note, these metabolites were also found in univariate analysis to be borderline-significantly increased in recurrers at week 1. At week 2, RF robustly identified lignoceroyl sphingomyelin as an important feature; this metabolite was also found to be significantly more abundant in recurrers in univariate analyses. RF also identified features with borderline significance that were found in the univariate analyses, including sphingomyelins, primary bile acids, and a phosphorylated lipid (Fig. 5). The predictive models also identified features that were not detected in univariate analyses: 4-hydroxyhippurate and bilirubin in the week 1 LR model were identified as predictive of recurrence when at higher levels. 4-hydroxyhippurate is a product of microbial degradation of polyphenols found in fruits and other plant-based foods [45]. Bilirubin is the product of host heme catabolism and is further reduced to urobilinoids/urobilinogens by the gut microbiome, so its higher levels in recurrers’ gut metabolomes is consistent with subpar microbiome function [36]. Because the predictive methods employed make different underlying assumptions (e.g., logistic regression is a generalized linear model whereas random forests is a nonlinear model), metabolites selected by multiple models are more likely to be robust [46]. Thus, the set of predictive metabolites identified by multiple methods (Fig. 5) may serve as strong candidates for future trials to validate biomarkers for recurrence prediction in larger, independent cohorts.

## Discussion

To our knowledge, our work represents the largest prospective study of CDI recurrence employing both microbiome sequencing and untargeted metabolomics analyses. Although prior studies have investigated some aspects of the relationship between the microbiome and CDI, our study design and analysis methods allowed us to probe further. The longitudinal nature of our study allowed us to investigate how rates of microbiome recovery relate to recurrence. Past studies investigating CDI recurrence were unable to observe the marked divergence of microbial diversity between recurrers and non-recurrers shortly after antibiotic treatment due to either a cross-sectional study design or a lack of systematic longitudinal sampling [15, 18–20]. Seekatz et al., though longitudinal, did not sample at the same timepoints for all participants; thus, although they noted that the alpha diversity of recurrers’ microbiomes trended lower than that of non-recurrers, they could not evaluate dynamic changes in alpha diversity within each group during the specific time-windows of interest [19]. Additionally, the prospective nature of our study allowed us to build *predictive* models of CDI recurrence, which are not possible with cross-sectional designs.

Moreover, by collecting broad gut metabolomic data, we were able to establish that this data can predict CDI recurrence more accurately than microbial composition data. The limited predictive capability of microbial sequencing data could be due to several factors, including lack of data about the status of host processes, poor sensitivity for detecting important low abundance organisms, and the inability to find common signal from diverse bacterial species that perform similar functional roles in the gut. However, it is also possible that predictive computational models specifically tailored to combining microbial compositional and metabolomic data could yield additional information and improve predictive accuracy.

Our findings have implications for design of diagnostic tests and therapeutic interventions for recurrent CDI. We did not find clear differences between recurrers and non-recurrers at the time of CDI diagnosis. Rather, we found that the rate of recovery from dysbiosis was substantially slower in recurrers, with incomplete recovery still evident at 2 weeks post-CDI antibiotic treatment. Further, we found that at 1 week post-CDI antibiotic treatment, increased levels of specific metabolite biomarkers associated with host inflammation accurately predicted future recurrence. Taken together, these findings suggest that diagnostic tests targeting specific metabolites in the first one to 2 weeks post-CDI treatment may be most accurate and clinically useful. Moreover, by identifying a small set of metabolites that accurately predict recurrence, we have laid the groundwork for developing a feasible clinical test based on a limited biomarker panel that could be cost-effectively measured through targeted LC/MS or other platforms that already exist in clinical laboratories.

Our study also uncovered complex and dynamic differences in gut metabolomes, both across time and between recurrers and non-recurrers, which could suggest new avenues for preventing or treating recurrent CDI. For example, we found increased levels of sphingomyelins, sphingolipids, and phospholipids in recurrers prior to the onset of symptoms. These lipids have been found in the guts of late-stage acute CDI in mice, as well as in children with IBD and CDI+IBD [10, 17], and may indicate early biomarkers of gut inflammation, as *C. difficile* begins to exert pathogenic effects that do not yet cause frank diarrhea. Interestingly, these lipids have recently been shown to be synthesized by common gut bacteria and affect vascular endothelium function and inflammatory responses [47, 48]. Thus, it is possible that rises in these metabolites seen in recurrers at least partially reflect metabolic activity of the microbiome, which could exacerbate development of CDI through modulation of host inflammatory and immune processes. Reduced endocannabinoids in recurrers could similarly involve an interplay between the host and microbiome, as recent evidence suggests that gut microbes regulate endocannabinoids in order to control energy metabolism and intestinal functions in the host [42].

While our study design and analysis methods produced results that have implications for better diagnostics and more effective treatments for recurrent CDI, our study did have several limitations that suggest opportunities for further research. Our study was conducted primarily within one hospital, and most participants were inpatients at the time the study commenced. These factors may partially explain why the recurrence rate in our parent study was 29%, which is higher than rates reported by some other studies, such as Guh et al. [3], which reported a 15% recurrence rate. However, other studies have reported higher or similar rates to our study. For example, Seekatz et al. [19] reported a 34% rate of recurrence among the 93 participants in their cohort, and Khanna et al. [18] reported a 28.5% rate of recurrence in their 88 participant cohort. The reasons for differences in CDI rates across studies is likely multifactorial, including differences in participant demographics and comorbidities. Another factor in differing CDI recurrence rates may be spatial-temporal trends possibly involving circulation of hypervirulent *C. difficile* strains within institutions and surrounding communities. Our study was conducted over approximately three years, a relatively short period of time, and did not assess *C. difficile* genetics.

An additional factor to be considered is that participants in our study received either vancomycin or metronidazole for initial CDI treatment. In our cohort, approximately 42% of recurrers received metronidazole versus 21% of non-recurrers. Although this difference was not statistically significant in our cohort, it was in the larger parent cohort [23], suggesting this effect was also present in our smaller cohort but failed to rise to the level of statistical significance due to the limited sample size. Indeed, metronidazole has previously been identified as a risk factor for CDI recurrence, and the updated Infectious Disease Society of America [49] clinical guidelines have removed metronidazole as a recommended first-line treatment for CDI for this reason. Additionally, vancomycin and metronidazole have entirely different mechanisms of action with known differential effects on the microbiome. When we controlled for antibiotic type used to treat initial CDI in statistical models, we still found many bacterial taxa and metabolites were significantly associated with recurrence/non-recurrence status. Further, we found that gut metabolites, but not antibiotic type used for initial CDI treatment, could significantly predict future CDI recurrence in statistical and machine learning models that accessed cross-validated performance. These findings suggest that there are significant differences between microbiomes of recurrers and non-recurrers that are not driven solely by differences in the antibiotic used for initial CDI treatment. However, higher order confounding between antibiotics, the gut environment and CDI recurrence are still a distinct possibility, and further studies will be important to clarify these effects.

Thus, follow up, multi-institutional studies that assess larger and more diverse cohorts receiving uniform vancomycin treatment for initial CDI, as well as integration of *C. difficile* genome analysis data will be important to generalize our findings. Another interesting question, which could not be addressed with our study design, is whether metabolite biomarkers reflect gut pathology that is permissive for CDI versus very early damage caused by CDI prior to the onset of symptoms. Ultimately, the true clinical value of CDI predictors, such as the metabolite biomarkers found in our study, will need to be assessed in the context of their utility for informing early interventions that significantly lower recurrence rates.

## Conclusions

We found in our prospective, longitudinal, multi-omic study of CDI recurrence that the gut microbial compositions and metabolomes of participants, while similar immediately before and after initial treatment, diverged rapidly as non-recurrers recovered normal microbiota and metabolic functions and recurrers remained dysbiotic. Our analyses uncovered specific metabolic derangements in participants who experienced subsequent recurrence, including evidence of loss of normal metabolic activities of the gut microbiome, host gut inflammation and cell death, and decreases in anti-inflammatory and immune-modulating compounds. Moreover, we found that differences in specific metabolites in the first 2 weeks post-CDI antibiotic treatment accurately predicted future recurrence, while microbiome sequencing data did not yield high predictive accuracy. These results suggest that metabolomics may be the more robust modality for evaluating recovery of microbial function. By providing specific candidate predictive biomarkers and expanding our knowledge of the complex metabolic changes preceding recurrence, our findings have implications for development of diagnostic tests and treatments for CDI recurrence.

## Methods

### Study design

Fecal samples analyzed for the present multi-omic study were collected as part of a larger prospective parent study that was conducted to assess predictors of CDI recurrence [23]. Participants with primary, uncomplicated CDI were identified by positive test results from the Brigham and Women’s Hospital (BWH) Clinical Microbiology Laboratory and recruited consecutively from BWH’s inpatient service, as well as two affiliated hospitals, between August 1, 2015 and September 1, 2018. Participants who were being treated for primary CDI, diagnosed with diarrhea symptoms and a positive *C. difficile* test by either glutamate dehydrogenase (GDH) or enzyme immunoassay (EIA) toxin or polymerase chain reaction (PCR), were eligible for inclusion. Primary CDI was defined as no episodes of CDI within the past 6 months. Exclusion criteria included inflammatory bowel disease, inherited or acquired immunodeficiencies, severe or fulminant CDI as defined by [49], or ongoing non-CDI antibiotic use that continued past the CDI antibiotic course.

The parent study followed 75 participants, 22 of whom recurred, from initial diagnosis until recurrence, or for 8 weeks post-treatment if they did not recur. Three participants were lost to follow-up. Samples were collected at diagnosis (if available), weekly or bi-weekly for 2 weeks after completion of anti-CDI antibiotics and then weekly for another 6 weeks, or until recurrence. Samples at diagnosis (before initiation of antibiotic treatment for CDI) were only available for some participants, because of the difficulty in obtaining viable fecal samples from the clinical laboratory workflow. Recurrence was defined as diarrhea (Bristol stool scale 6 or 7), at least 3 bowel movements daily for 3 days, and a positive GDH and EIA test, in keeping with current guidelines.

Participants for the multi-omic study were chosen from the parent study based on the availability of a week 1 stool sample, a desired ratio of approximately 2:1 non-recurrers to recurrers to sufficiently power predictive analyses while maximizing study resources, and age and sex matching between non-recurrers and recurrers. This yielded a cohort of 34 non-recurrent and 19 recurrent participants for the multi-omic study. Because all participants recurred before week 4, samples after week 4 were not analyzed.

### Participant demographic and clinical data

Weight and height were collected individually and used to calculate participants’ BMI. Significance testing for demographic and clinical variables was conducted using Fisher’s exact test for binary variables, the chi-squared test for categorical variables, and the Wilcoxon-rank-sum test for continuous variables. To ensure a sufficient number of participants for valid statistical inference, significance testing was only performed on demographic/clinical variables if greater than 3 participants had the characteristic of interest.

### 16S rRNA gene amplicon sequencing

For DNA extraction, all fecal samples were processed with the Zymo Research ZymoBIOMICS DNA 96-well kit according to manufacturer instructions with the addition of bead beating for 20 min. The extracted DNA was used for 16S rRNA gene Amplicon sequencing and 16S rRNA qPCR for total bacterial concentration estimation. Amplicon sequencing of the v4 region of the 16S rRNA gene was performed using the previously described protocol in [50] using 515F and 806R primers for PCR along with:

- 5′-[Illumina adaptor]-[unique bar code]-[sequencing primer pad]-[linker]-[primer]
- Read 1 (forward primer): AATGATACGGCGACCACCGAGATCTACAC-NNNNNNNN-TATGGTAATT-GT-GTGCCAGCMGCCGCGGTAA
- Read 2 (reverse primer): CAAGCAGAAGACGGCATACGAGAT-NNNNNNNN-AGTCAGTCAG-CC-GGACTACHVGGGTWTCTAAT

### LC-MS untargeted metabolomics

LC-MS untargeted metabolomics was performed by Metabolon (Durham, NC USA). After delivery to Metabolon, samples were homogenized in methanol to extract metabolites and then centrifuged to separate the supernatant from debris and precipitates. The supernatant was divided into five aliquots for four analyses plus one extra and then dried using a TurboVap (Zymark). Dried samples were stored overnight under nitrogen gas. Samples were reconstituted and measured with Waters ACQUITY ultra-performance liquid chromatography (UPLC) and Thermo Scientific Q-Exactive high resolution/accurate mass spectrometry (MS), heated electrospray ionization source (HESI-II) and Orbitrap pass analyzer (35,000 mass resolution). Samples were analyzed in the following four different ways: (1) elution with C18 column (Waters UPLC BEH C18-2.1 × 100 mm, 1.7 μm) in positive-ion mode with water/methanol gradient mobile phase containing 0.05% perfluorpentanoic acid (PFPA) and 0.1% formic acid (FA), (2) as in (1), except with water/acetonitrile/methanol gradient mobile phase containing 0.05% PFPA and 0.01% FA, (3) elution with a separate C18 column in negative-ion mode with water/methanol gradient mobile phase containing 6.5 mM ammonium bicarbonate, pH 8, and (4) elution with HILIC column (Waters UPLC BEH amide 2.1 × 150 mm, 1.7 μm) in negative-ion mode with water/acetonitrile gradient mobile phase containing 10 mM ammonium formate, pH 10.8. The MS analysis was performed as dynamic exclusion, altering between MS and data-dependent MS^n^ scans with a scan range of 70–1000 m/z. Data extraction, peak identification, quality control, and annotation were performed by Metabolon’s proprietary software.

### Short chain fatty acid profiling

Volatile SCFAs were quantified as described in [51]. Acidified internal standards with 100uL of either ethyl anhydrous or boron trifluoride-methanol was added to 100 μL of supernatant from homogenized cecal contents. Chromatographic analyses were carried out on an Agilent 7890B system with a flame ionization detector (FID). Chromatogram and data integration were done using the OpenLab ChemStation software (Agilent Technologies, Santa Clara, CA). SCFAs were identified by comparing their specific retention times relative to the retention time in the standard mix. Concentrations were determined as mM of each SCFA per gram of sample for the raw cecal/fecal material. The Agilent platform cannot discriminate between isovalerate and 2-methylbutyrate, and so these are reported as a single peak value.

### 16S rRNA gene amplicon data analysis

#### Bioinformatics

Sequencing generated 4,605,740 total reads and 97,994 average reads per sample. The paired-end Fastq files were truncated, filtered, denoised, and merged using the dada2 pipeline and filtering parameters identical to the dada2 tutorial [52]. Our analysis found 2509 unique amplicon sequencing variants (ASVs) in the dataset, and taxonomy was assigned using the dada2 RDP and Silva reference databases [53, 54]. If taxa assignments between the two databases disagreed, the taxa assignment from the RDP database was used.

#### Alpha and beta diversity

Prior to calculating alpha and beta diversity, relative ASV abundance was calculated by dividing each ASV’s counts by the total number of counts in a sample. Using ASV relative abundance, we calculated the alpha diversity (Chao1) at pre-treatment, week 1, week 2, week 3, and week 4 using scikit-bio (skbio.diversity.alpha.chao1) [26]. Significant differences in intra-group alpha diversity over time and inter-group alpha diversity at pre-treatment, week 1, and week 2 were tested using the Mann-Whitney *U* test. A one-sided test was used to test the hypothesis that alpha diversity of both groups decreased during antibiotic treatment and then recovered, and the hypothesis that non-recurrent participants would have higher alpha diversity. Beta diversity was calculated at pre-treatment, week 1, week 2, week 3, and week 4 from the Bray-Curtis dissimilarities (calculated using scipy.spatial.distance.pdist) of relative ASV abundances between each subject. To visualize the dissimilarity of outcome groups at each timepoint and the intra-group dissimilarity between timepoints, we performed multi-dimensional scaling (using scikit-learn.manifold.MDS) on the Bray-Curtis dissimilarities. We used permutational multivariate analysis of variance (PERMANOVA) (skbio.stats.distance.permanova) with 999 permutations to assess the significance of inter- and intra-group dissimilarities at pre-treatment, week 1, and week 2.

#### Filtering

Prior to differential abundance analysis, ASVs were filtered to remove rare taxa. We included ASVs present with > 10 counts and in ≥ 10% of participants in either pre-treatment, week 1, or week 2. This resulted in 237 ASVs post-filtering.

### Differential abundance analysis

After filtering, differential abundance analyses between recurrers and non-recurrers at pre-treatment, week 1, and week 2 were performed using the DESeq function within the DESeq2 package [55]. To control for effects of CDI treatment with either vancomycin or metronidazole, antibiotic treatment type was included as a covariate in the regression equation for inter-group analyses at week 1 and week 2. Because every ASV in the dataset contained zeros, we pre-computed the geometric means and then the size factors using the estimateSizeFactors function within DESeq2. Intra-group differential abundance analysis was also performed between pre-treatment and week 1, and between week 1 and week 2, for both recurrers and non-recurrers using the same procedure in DESeq2, including controlling for the antibiotic treatment type. All differential abundance analyses were followed by the Benjamini-Hochberg correction for multiple hypotheses [56]. The relative abundances of ASVs that were significantly different between recurrers and non-recurrers at pre-treatment, week 1, or week 2 are shown in Fig. 2 on a logarithmic scale, along with the phylogenetic relationships of these ASVs (found with methods detailed below). In this figure, recurrers and non-recurrers at each timepoint are clustered hierarchically using scipy.cluster.hierarchy with optimal ordering and ‘average’ distance.

### Phylogenetic placement

To further clarify phylogenetic relationships between ASVs of interest, we built a reference tree and then performed phylogenetic placement of ASVs. For the reference tree, all typed, isolated strains of good quality that were longer than 1200 base pairs were downloaded from the RDP bacteria and archaea datasets [53]. Reference sequences were then aligned using the RDP aligner. The reference sequences were then filtered to remove: (1) sequences with unaligned lengths ≥ 1600 bp and, (2) sequences with rare insertions (defined as a base pair in a position where there were 5 or less sequences with un-gapped base pairs in that position). Filtered reference sequences were then realigned using the same RDP aligner. A reference tree was constructed using FastTree version 2.1.7 SSE3 with the general-time-reversible maximum likelihood option [57]. Pplacer v1.1.alpha19 with default settings [58] was then used to place query ASVs onto the reference tree.

### Enrichment analysis

Enrichment analyses were performed on the ASVs found in each differential abundance analysis with FDRs < 0.05 (Additional file 5). For a given family A, we tested if the family was significantly overrepresented in differentially abundant ASVs using the hypergeometric probability distribution:

1 $$P\left(X=k\right)=\frac{\left(\begin{array}{c}K\\ {}k\end{array}\right)\left(\begin{array}{c}N-K\\ {}n-k\end{array}\right)}{\left(\begin{array}{c}N\\ {}n\end{array}\right)}$$

Here, *N* is the total number of (post-filtering) ASVs, *K* is the subset of *N* in family A, *n* is the number of differentially abundant ASVs, and *k* is the subset of *n* in family A. To prevent false positives due to small family sizes, we did not test (1) families that had too few ASVs in the total post-filtering set (*K* ≤ 3) or (2) families that had too few ASVs in the differentially abundant subset (*k* ≤ 2). For all families large enough to pass the filter, *p* values were computed using the hypergeometric test, and the Benjamini-Hochberg procedure was using to correct for multiple hypothesis testing [56].

### LC-MS untargeted metabolomics data analysis

“OrigScale” data returned by Metabolon was used in all analyses described in this manuscript; these data represent values normalized in terms of raw area counts.

### Ordination analyses

To assess inter-group dissimilarity at each timepoint and intra-group dissimilarity between timepoints, we computed matrices using Spearman rank correlation on the unfiltered and untransformed metabolomic data. We used PERMANOVA (skbio.stats.distance.permanova) with 999 permutations to test the significance of differences (Additional file 3).

### Filtering

LC-MS untargeted metabolomics measured 1387 metabolites. To retain only metabolites with sufficient prevalence and variance across time or participants, we included metabolites with: (1) non-zero values in ≥ 25% of participants in either pre-treatment, week 1, or week 2 samples, and (2) co-efficient of variations in the top 50th percentile in either pre-treatment, week 1, or week 2 samples. These criteria resulted in 760 metabolites post-filtering.

### Univariate analysis

Before univariate analyses, metabolite values were log transformed (after adding a small positive number, 10% of the minimum non-zero value in the dataset) to all values, and standardized to have a mean of 0 and a standard deviation of 1. After filtering and transforming the metabolic data, we performed ordinary least squares linear regression using the statsmodels (v0.14.0) package in python, with both antibiotic treatment type (metronidazole or vancomycin) and recurrence/non-recurrence as covariates. To control for the effect of antibiotic type on the level of each metabolite, hypothesis testing was performed using the *t*-test on the coefficient of the recurrence/non-recurrence variable. The Benjamini-Hochberg procedure was applied to correct for multiple hypothesis testing [56].

### Enrichment analysis

Enrichment analyses were performed on the metabolites found in univariate analysis with FDRs < 0.05, with an analogous method as used for enrichment analysis of the ASVs. We used the hypergeometric test with Benjamini-Hochberg [56] multiple hypothesis correction to assess whether pre-specified groups (super pathways and sub-pathways) were significantly over-represented in the differentially abundant metabolites. As with the ASV enrichment analysis, we did not perform hypothesis testing on super and sub-pathways with (1) too few metabolites in the total post-filtering set (*K* ≤ 3) or, (2) too few metabolites in the differentially abundant subset (*k* ≤ 2).

### Short-chain fatty acid data analysis

SCFA profiling found eight SCFAs in the analyzed samples: acetate, propionate, isobutyrate, butyrate, isovalerate/2-methylbutyrate, valerate, heptanoate, isocaproate, and caproate. Heptanoate was removed from subsequent analyses due to only being present in one sample. Caproate was present in only two samples and was also removed from further analyses. The remaining seven SCFAs were log transformed and standardized analogously to the untargeted metabolomics data prior to univariate analysis; univariate analyses were also performed analogously to those on the untargeted metabolomics data.

### Predictive modeling

The following data sources were used as input to predictive modeling methods: (1) clinical variables found to be associated with recurrence in prior studies (age, previous PPI use, antibiotic treatment regiment, and CDI diagnostic test), (2) 16S rRNA amplicon sequencing (ASVs) from pre-treatment, week 1, or week 2 samples, (3) untargeted metabolomics data from pre-treatment, week 1, or week 2 samples, (4) SCFA profiles from pre-treatment, week 1, or week 2 samples, and (5) data sources 1–4 combined. In each predictive model, training datasets were filtered with the same criteria described for univariate analyses. Metabolites and SCFAs were log-transformed and standardized, and ASV relative abundances were transformed with the centered log ratio and then standardized. Continuous clinical variables (i.e., age) were log-transformed and standardized.

Relevant predictive features were identified through a nested leave-one-out cross-validation procedure (described in detail below for each method). To summarize the results for each feature, we report the median and 95% interval over the folds (i.e., regression coefficients for logistic and cox regression, feature importances for random forests). We deem features significant if the 95% cross-validated odds-ratio/feature-importance intervals did not contain 1.0, and marginally significant if the 75% cross-validated interval did not contain 1.0. The code to reproduce these analyses can be found in https://github.com/gerberlab/cdiff_paper_analyses.

### Logistic regression

Logistic regression models were fit using scikit-learn’s (v0.24.2) logistic regression function with L1 lasso regularization, balanced classes, and a liblinear solver. We used nested leave-one-out cross validation to find the optimal L1 lambda hyperparameter, performing a grid search over a range of 200 values from the maximum lambda value (i.e., the value that resulted in all zero coefficients) to 0.1% of the maximum lambda value. Performance in the inner loop was evaluated by area under the receiver operator curve (AUC) score calculated from the predictions of all the held-out samples. To reduce overfitting, the inner loop performances were smoothed using a *n* = 5 moving average, and the optimal L1 hyperparameter was that which resulted in the highest value on the smoothed performance curve. After choosing the best L1 hyperparameter, the model’s predictive capability was evaluated by its leave-one-out cross validated AUC score. Variance estimates of model performance and regression coefficients were calculated from the cross-validation folds.

### Random forest

Random forest models were fit using scikit-learn’s (v0.24.2) random forest classifier. We performed a nested leave-one-out cross validation procedure with grid search, to determine the number of estimators (50 or 100), the maximum features to subsample at each split (the total number of features or the square root of the number of features), the minimum samples required to split an internal node (2 or 9) and the minimum samples required to split a leaf node (1 or 5). All other parameters were set to their default values except for class weight (‘balanced’) and out of box score (True). The feature importances were calculated with the impurity-based feature importance, or the Gini importance, using the *feature_importance* attribute of the fitted model. Model performance and feature importance statistics were calculated from the cross-validation folds.

### Cox regression

Cox regression models were fit using scikit-survival’s (v0.15.0) Coxnet Survival Analysis function with L1 regularization. We used a similar nested cross validation as described for our logistic regression analyses to optimize the L1 lambda parameter, searching over a range of 200 values from the maximum lambda value (i.e., the value that resulted in all zero coefficients) to 0.01% of the maximum lambda value. We evaluated both the inner and outer loops of the survival analysis using the concordance index (CI). Rather than leave-one-out cross validation, we used a leave-two-out method, where all left out pairs had at least one recurrer, to calculate the CI. In this formulation (mathematically equivalent to the standard definition of CI), CI is computed by dividing the number of times a pair was ordered correctly by the number of times a pair ordering was attempted. Variance estimates of model performance were calculated from the cross-validation folds.

## Supplementary Information

**Additional file 1.** Participant co-morbidities: provides sufficient statistics and statistical testing for co-morbidities experienced by more than 3 participants.

**Additional file 2.** Ecological diversity analyses for 16S rRNA gene amplicon sequencing data, and ordination analyses for metabolomic data: provides the sufficient statistics and statistical testing for alpha and beta diversity analyses for 16S rRNA gene sequencing data, as well as analogous information for the ordination analyses of the metabolomics data.

**Additional file 3.** Univariate statistical analyses for 16S rRNA gene amplicon sequencing, short-chain fatty acids, and metabolomic data: provides the results of univariates statistical analyses on ASVs, metabolites, and SCFAs.

**Additional file 4.** Taxonomic enrichment analyses for sequencing data, and pathway enrichment analyses for metabolomic data: provides the results of enrichment analyses done to determine if the set of features found to be significant in univariate analysis were significantly enriched for a bacterial family or metabolite category.

**Additional file 5.** Predictive modeling results: provides the results of the predictive analyses run for each data source at pre-treatment, week 1, and week 2.

**Additional file 6.** Predictive modeling feature analysis: provides the features identified in predictive analyses that were found to affect the odds of recurrence in logistic regression or cox regression, or to be identified as important features in random forest analyses.

**Additional file 7: Figure S1.** Microbiome community structure significantly changed within groups and significantly differed between groups at week two. Beta diversity with the Bray-Curtis dissimilarity measure was used to assess overall gut microbiome community structure; Principal Coordinate Analysis (PCoA) was used to visualize results. (A) Beta diversity changed significantly over time within groups. Differences were significant for non-recurrers from pre-treatment to week one (*p* = 10^-3^) and from week one to week two (*p* = 10^-3^). For recurrers, differences were significant from pre-treatment to week one (*p* = 3x10^-3^). (B) Beta diversity was significantly different between recurrers and non-recurrers at week two (*p* = 10^-2^); differences at other time-points were not significant.

**Additional file 8: Figure S2.** Gut metabolome structure significantly changed within groups and significantly differed between groups at week two. Ordination analysis using Spearman rank correlation was used to assess overall metabolome structure; Principal Coordinate Analysis (PCoA) was used to visualize results. (A) Metabolome structure changed significantly over time within groups. Differences were significant for non-recurrers from pre-treatment to week one (*p* = 10^-3^) and from week one to week two (*p* = 10^-3^). For recurrers, differences were significant from pre-treatment to week one (*p* = 10^-3^). (B) Metabolome structure was significantly different between recurrers and non-recurrers at week two (p=10^-3^); differences at other time-points were not significant.

**Additional file 9: Figure S3.** Borderline significantly higher fecal acetate and isovalerate SCFAs were observed at week two in non-recurrers. Log-transformed and standardized concentrations of the short-chain fatty acids (SCFAs) measured in fecal samples are shown. Levels of acetate (FDR = 0.07) and isovalerate/2-ME butyrate (FDR = 0.07) were higher in non-recurrent (NR) versus recurrent (R) participants.

## Data Availability

The sequencing datasets generated and/or analyzed during the current study are available in the SRA repository, accession number PRJNA772946, https://dataview.ncbi.nlm.nih.gov/object/PRJNA772946. Processed sequencing data as well as untargeted metabolomics data and targeted SCFA data can be accessed in Zenodo at 10.5281/zenodo.6473881. More detailed results of univariate and predictive analysis than in the additional files can also be accessed at the above link, as well as data needed to reproduce figures and analyses. Data needed to reproduce figures from predictive analyses without re-running the analyses can be accessed at 10.5281/zenodo.5703429. All other datasets supporting the conclusions of this article are included within the article and its additional files. Code to reproduce the analysis and figures in this paper is publicly available in the Github project cdiff_paper_analyses at https://github.com/gerberlab/cdiff_paper_analyses. Code is platform independent and written in python (3.7.10) and R (4.1.0). All R and python packages and version numbers can be found in the Github repo readme.md file.
