# Supplementary figures and images for: Gut metabolites predict Clostridioides difficile recurrence
Source: Microbiome. 2022 Jun 9;10:87. doi: 10.1186/s40168-022-01284-1 (PMC9178838; doi:10.1186/s40168-022-01284-1)

**A**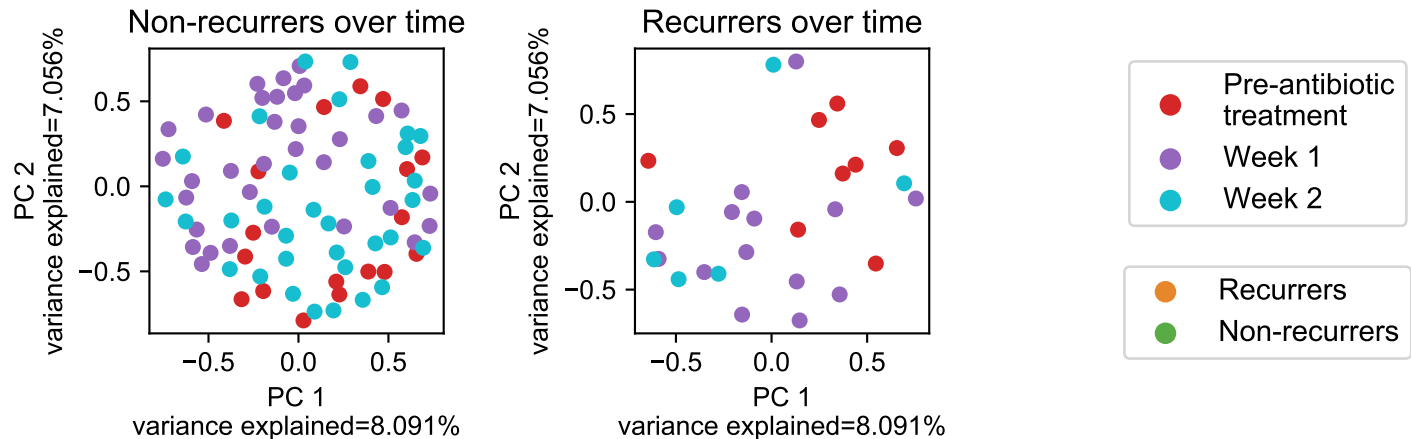**B**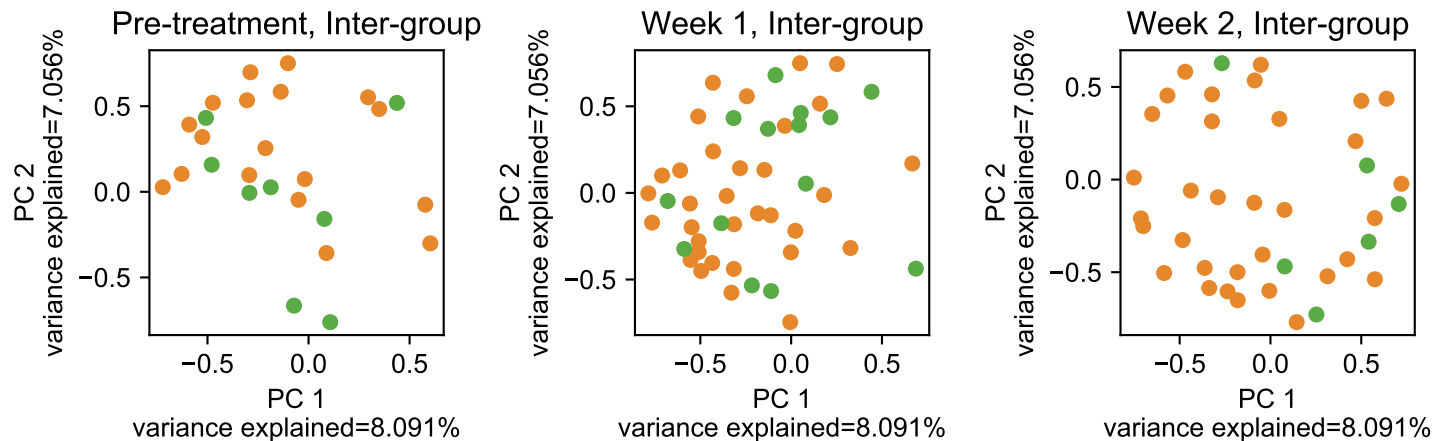

Supplement: Supplementary file 8 — Additional file 7: Figure S1. Microbiome community structure significantly changed within groups and significantly differed between groups at week two. Beta diversity with the Bray-Curtis dissimilarity measure was used to assess overall gut microbiome community structure; Principal Coordinate Analysis (PCoA) was used to visualize results. (A) Beta diversity changed significantly over time within groups. Differences were significant for non-recurrers from pre-treatment to week one (p = 10-3) and from week one to week two (p = 10-3). For recurrers, differences were significant from pre-treatment to week one (p = 3x10^-3^). (B) Beta diversity was significantly different between recurrers and non-recurrers at week two (p = 10-2); differences at other time-points were not significant. [file 40168_2022_1284_MOESM7_ESM.pdf]

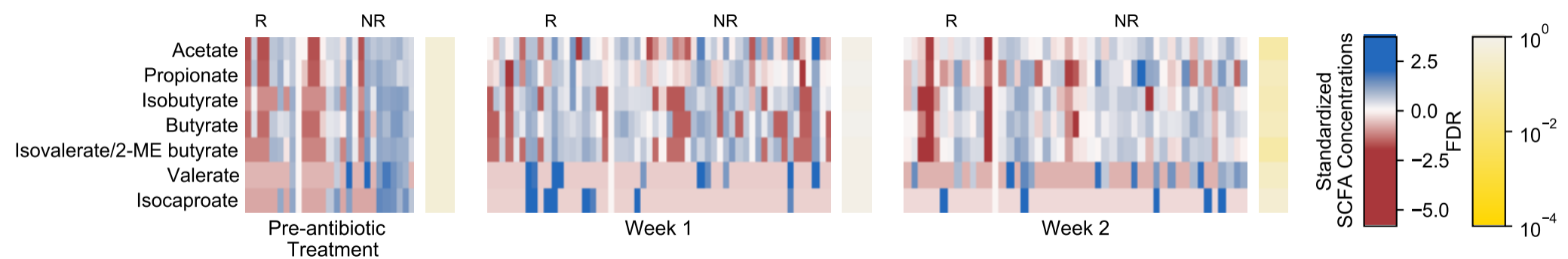

Supplement: Supplementary file 10 — Additional file 9: Figure S3. Borderline significantly higher fecal acetate and isovalerate SCFAs were observed at week two in non-recurrers. Log-transformed and standardized concentrations of the short-chain fatty acids (SCFAs) measured in fecal samples are shown. Levels of acetate (FDR = 0.07) and isovalerate/2-ME butyrate (FDR = 0.07) were higher in non-recurrent (NR) versus recurrent (R) participants. [file 40168_2022_1284_MOESM9_ESM.pdf]
