# Supplementary material for: Gut metabolites predict Clostridioides difficile recurrence
Source: Microbiome. 2022 Jun 9;10:87. doi: 10.1186/s40168-022-01284-1 (PMC9178838; doi:10.1186/s40168-022-01284-1)
Supplement: Supplementary file 9 — Additional file 8: Figure S2. Gut metabolome structure significantly changed within groups and significantly differed between groups at week two. Ordination analysis using Spearman rank correlation was used to assess overall metabolome structure; Principal Coordinate Analysis (PCoA) was used to visualize results. (A) Metabolome structure changed significantly over time within groups. Differences were significant for non-recurrers from pre-treatment to week one (p = 10-3) and from week one to week two (p = 10-3). For recurrers, differences were significant from pre-treatment to week one (p = 10-3). (B) Metabolome structure was significantly different between recurrers and non-recurrers at week two (p=10-3); differences at other time-points were not significant. [file 40168_2022_1284_MOESM8_ESM.pdf]

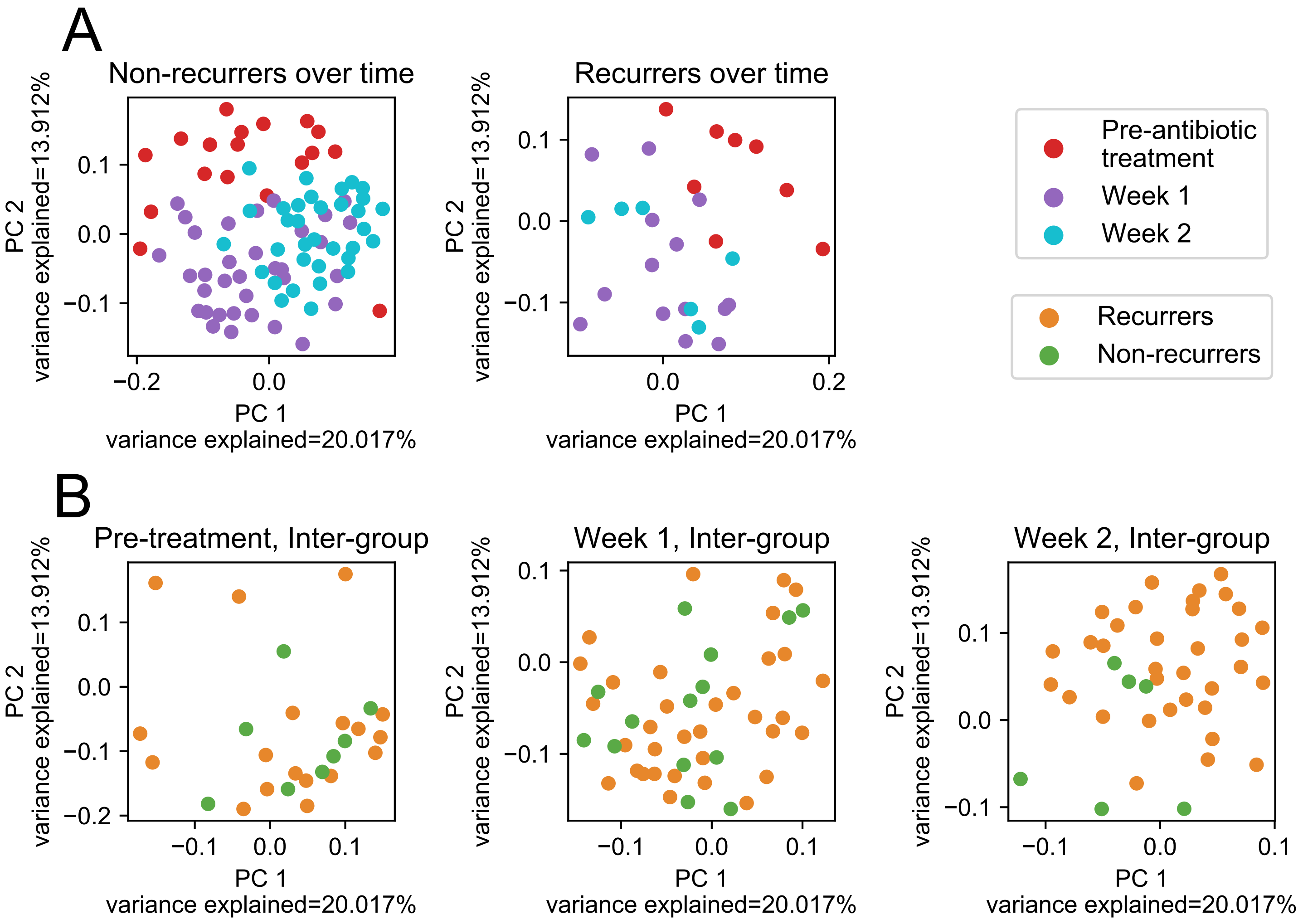
A
Non-recurrers over time
Recurrers over time
Pre-antibiotic treatment
Week 1
Week 2
Recurrers
Non-recurrers
B
Pre-treatment, Inter-group
Week 1, Inter-group
Week 2, Inter-group
PC 1
variance explained=20.017%
PC 2
variance explained=13.912%
